# Persistent mTORC1 activation underlies sex dimorphic progression of MASLD in mice with hepatocyte prohibitin-1 deficiency

**DOI:** 10.21203/rs.3.rs-7481614/v1

**Published:** 2025-09-25

**Authors:** Amany A. Alowaisi, Jolonda C. Mahoney, Ran Huo, Islam A. Berdaweel, Rachel A. Crawford, Kendall J. Mallaro, Jared M. McLendon, Ethan J. Anderson

**Affiliations:** Department of Pharmaceutical Sciences & Experimental Therapeutics, College of Pharmacy, University of Iowa, Iowa City, IA, U.S.A.; Department of Clinical Pharmacy, College of Pharmacy, Yarmouk University, Irbid, Jordan; Department of Pharmaceutical Sciences & Experimental Therapeutics, College of Pharmacy, University of Iowa, Iowa City, IA, U.S.A.; Department of Pharmaceutical Sciences & Experimental Therapeutics, College of Pharmacy, University of Iowa, Iowa City, IA, U.S.A.; Department of Pharmaceutical Sciences & Experimental Therapeutics, College of Pharmacy, University of Iowa, Iowa City, IA, U.S.A.; Department of Clinical Pharmacy, College of Pharmacy, Yarmouk University, Irbid, Jordan; Department of Pharmaceutical Sciences & Experimental Therapeutics, College of Pharmacy, University of Iowa, Iowa City, IA, U.S.A.; Department of Pharmaceutical Sciences & Experimental Therapeutics, College of Pharmacy, University of Iowa, Iowa City, IA, U.S.A.; Department of Pharmaceutical Sciences & Experimental Therapeutics, College of Pharmacy, University of Iowa, Iowa City, IA, U.S.A.; Abboud Cardiovascular Research Center, Carver college of Medicine, University of Iowa, Iowa City, IA, U.S.A.; Department of Pharmaceutical Sciences & Experimental Therapeutics, College of Pharmacy, University of Iowa, Iowa City, IA, U.S.A.; Abboud Cardiovascular Research Center, Carver college of Medicine, University of Iowa, Iowa City, IA, U.S.A.; Fraternal Order of Eagles Diabetes Research Center, Carver College of Medicine, University of Iowa, Iowa City, IA, U.S.A.

## Abstract

Prohibitins (PHB1,2) are highly conserved lipid-raft associated proteins that physically interact to form a multimeric ring supercomplex in mitochondrial and plasma membranes where they are intimately involved in regulating cellular metabolism. Prior studies in disparate cell models have implicated PHB1 as a mediator of insulin signaling and its downstream effector, the mechanistic target of rapamycin complex 1 (mTORC1), but the mechanisms and physiological implications of these interactions are unclear. Here, we examined the role of PHB1 in regulating insulin and nutrient mediated activation of mTORC1 in liver using genetic and pharmacological approaches in mice and hepatocyte culture. Interestingly, male mice with hepatocyte-specific PHB1 haploinsufficiency (hPHB1-KD) at 6 months displayed features consistent with metabolic dysfunction-associated steatotic liver disease (MASLD), characterized by liver steatosis and impaired glucose tolerance with hyperinsulinemia, while these parameters were unaffected or even mildly improved in age-matched hPHB1-KD females. Both sexes of hPHB1-KD mice displayed increased basal phosphorylation of mTORC1 and its downstream targets (S6, 4EBP1) in liver compared with WT in fasted state, with minimal responsiveness to insulin. Transcriptomic data revealed strong upregulation of *Lpin1* gene in male hPHB1-KD mice, a phosphatidic acid phosphatase regulated by mTORC1 that critically regulates hepatic lipid metabolism. Integrated transcript-/metabolomic analysis showed enriched glycerolipid metabolism and upregulation of MASLD pathway in the liver of hPHB1-KD males. Parallel experiments in AML12 hepatocytes confirmed that PHB1 knockdown causes hyper-activation of mTORC1 signaling, increased cytoplasmic lipin-1 expression and localization, and increased lipid droplet formation. Furthermore, one week of treatment with mTORC1 inhibitor Torin1 reduced hepatic triglycerides and normalized mTORC1 signaling in hPHB1-KD males to levels comparable with WT. Collectively, these findings demonstrate that PHB1 is essential for maintaining metabolic homeostasis in liver via control of mTORC1-lipin1 axis, and further confirm that metabolic effects of PHB1 deficiency in liver are sexually dimorphic.

## Introduction

Among the most highly conserved proteins in nature are the prohibitin domain containing family of proteins (1, 2). Prohibitin-1 (PHB1) is a pleiotropic, evolutionarily conserved protein ubiquitously expressed in all eukaryotic cells that, depending on cell type and intracellular localization, participates in a variety of cellular functions including proliferation, metabolism, and apoptosis (3, 4). Owing to these diverse functions, PHB1 has gained significant attention as a potential therapeutic target in chronic diseases such as obesity, type 2 diabetes, neurodegenerative diseases and cancer (5, 6). PHB1 associates with its closely related homolog prohibitin 2 (PHB2) in mitochondrial membranes, forming a large ring-like complex that functions as a mitochondrial chaperone to preserve mitochondria structure and function (7, 8). Loss of either PHB1 or PHB2 via genetic ablation results in destabilization and equivalent ablation of the other, highlighting PHB1/2 interdependence within the mitochondria at the protein level. However, their functional roles in extra-mitochondrial compartments appear to be independent and non-redundant (9).

PHB1 has an essential role in preserving liver integrity and function, and its deficiency is linked to liver injury (3, 10, 11). Complete ablation of hepatocyte PHB1 quickly leads to liver cholestatic injury, steatohepatitis, and carcinogenesis (3). Moreover, reduced PHB1 levels have also been reported in livers of obese mice and in obese patients who are at increased risk of metabolic associated steatohepatitis (MASH) (12). Recent studies have implicated PHB1 as a potential mediator of two major nutrient-sensing kinases in the liver, AMPK and mTORC1 (13, 14). Specifically, mechanistic target of rapamycin complexes 1 and 2 (mTORC1,2) signaling coordinates hepatocellular growth and metabolism, and loss of mTORC1 regulation contributes to pathogenesis of metabolic dysfunction associated steatotic liver disease (MASLD) (15, 16). Through its activation by nutrients, insulin and growth factor signaling (17), mTORC1 enhances protein synthesis via phosphorylation of key downstream targets such as ribosomal S6 kinase (S6) and eukaryotic initiation factor 4E-binding protein (4EBP) (18). Activated mTORC1 also promotes lipogenesis through activation of sterol regulatory element-binding protein 1 (SREBP1), which regulates lipogenic gene expression, either via S6 (19) or phosphorylation and cellular localization of its downstream target, lipin-1 (17, 20). Further understanding of the molecular mediators and regulators within the mTORC1 axis in liver is therefore essential for identifying new therapeutic targets in metabolic diseases.

Interestingly, PHB1 ablation in Schwann cells was found to cause constitutive mTORC1 activation, leading to demyelination and peripheral neuropathy in mice (21). Another report showed that PHB2 knockdown in podocytes also led to mTORC1 hyper-activation and breakdown of the kidney filtration barrier (22). Despite the drastic difference between the cell models used in those studies and hepatocytes, it seemed plausible that a link between PHB’s and mTORC1 might underlie insulin responsiveness and control of metabolic homeostasis by the liver. In this study, we generated a constitutive hepatocyte-specific PHB1 haploinsufficient (i.e. heterozygous) mouse model to investigate these questions. The rationale for using a partial PHB1 knockdown model is two-fold: first, homozygous PHB1 knockout in hepatocytes is severely toxic and rapidly leads to hepatocellular carcinoma as previously described (11), and second, prior studies have reported reduced liver PHB1 levels in obese patients at risk of MASH (12), thereby increasing translational significance of a PHB1 deficient model.

Our findings reveal an important role for PHB1 in regulating mTORC1 activity and lipogenesis in hepatocytes, in part via control of lipin-1 expression, and further show that loss of PHB1 levels leads to MASLD in a sex-specific manner.

## Results

### Hepatocyte PHB1 deficiency does not significantly affect liver mitochondrial function but promotes steatosis in male mice

**Figure 1:**
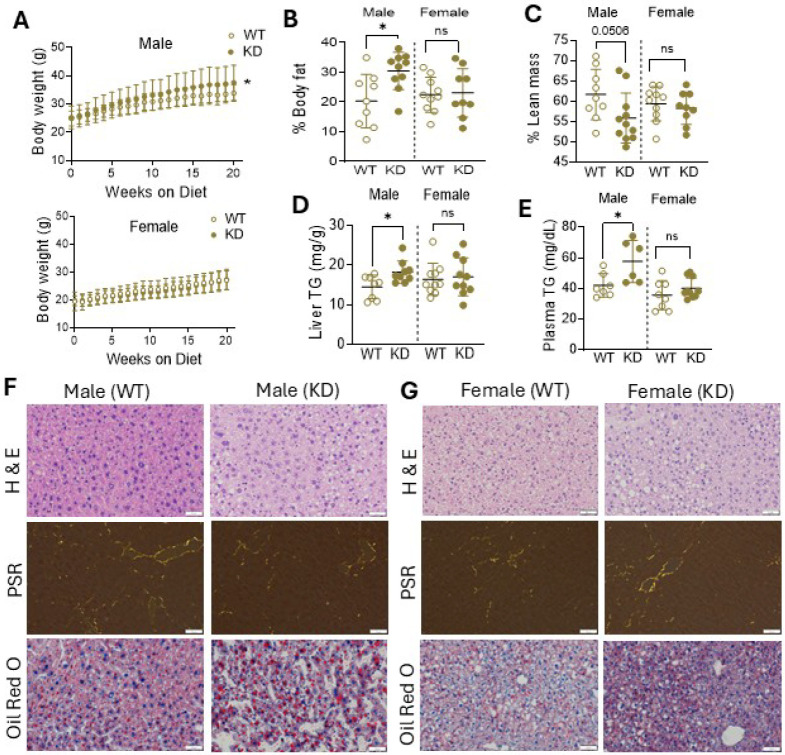
Impact of hepatocyte PHB1 deficiency on body composition and triglyceride metabolism. **A.** Mice body weight changes over the duration of the study (n=12/group) in WT and hPHB1-KD male (top panel) and female (lower panel) mice. **B**. Body fat and lean mass **(C)** percents of WT and hPHB1-KD male and female mice determined by NMR analysis at 6 months age (n=6–12/group). **D**. Triglycerides level in Liver tissue homogenates and plasma **(E)** of WT and hPHB1-KD male and female mice (n=6–10/group). **F**. Representative images showing hematoxylin and eosin (H&E), picrosirius red (PSR) and oil red O staining of histological sections of liver tissues from WT and hPHB1-KD male (left panel) and **(G)** female (right panel) mice (scale bar 50 μm), n=2–4/group, images are representative of 10 image fields captured per mouse under X20 magnification. All data are presented as mean SD. *P<0.05.

To determine the role of PHB1 in liver metabolic regulation, we generated hepatocyte-specific PHB1 deficient mice (hPHB1-KD) by crossing *Phb1*^*fl/fl*^ mice with mice expressing Cre recombinase under control of the albumin promoter (Figure S1A, B). Only heterozygous mice (i.e. deletion of one *Phb1* allele) were included in this study. We confirmed the knockdown of PHB1 in liver tissues at both the mRNA and protein levels (Figure S1C, D). Consistent with previous findings, the reduction in PHB1 was accompanied by a decrease in PHB2, highlighting their interdependence for protein stability. Both male and female mice were included in our study as previous reports have found that PHB1 has metabolic effects that are sex specific (23). Mice were maintained on a regular chow diet for 16 weeks, and final testing performed at 5–6 months of age. Male hPHB1-KD mice had a significantly higher body weight compared with WT mice at end of the study (P < 0.05); an effect not observed in female hPHB1-KD mice (Fig. 1A). This increased body weight was accompanied by greater % body fat and lower lean mass in male hPHB1-KD mice compared with WT, whereas no comparable differences were observed in female hPHB1-KD mice (Fig. 1B, C). Triglyceride levels in liver and plasma were also increased in hPHB1-KD males compared with WT (Fig. 1D, E). Histological analysis with oil red O staining revealed larger lipid droplets in hPHB1-KD males, but no significant liver structural injury or fibrosis as shown in hematoxylin and eosin (H & E) and picrosirius red (PSR) staining, respectively (Fig. 1F). No lipid droplet accumulation was observed in livers of female hPHB1-KD mice (Fig. 1G).

As steatosis is frequently a consequence of decreased mitochondrial content and/or fat oxidation capacity, mitochondrial respiration was assessed in fresh liver samples from WT and hPHB1-KD mice using a variety of substrates. No significant differences in maximal ADP-stimulated respiration were observed with any substrate in either male or female hPHB1-KD mice, although males had lower basal (i.e., no ADP, state 2) respiration with palmitoyl-l-carnitine (Figure S2A). Further assessment of mitochondrial respiratory complex expression in liver lysates revealed no differences between WT and hPHB1-KD mice (Figure S2B).

### Systemic glucose metabolism is perturbed in male mice with hepatocyte PHB1 deficiency

**Figure 2:**
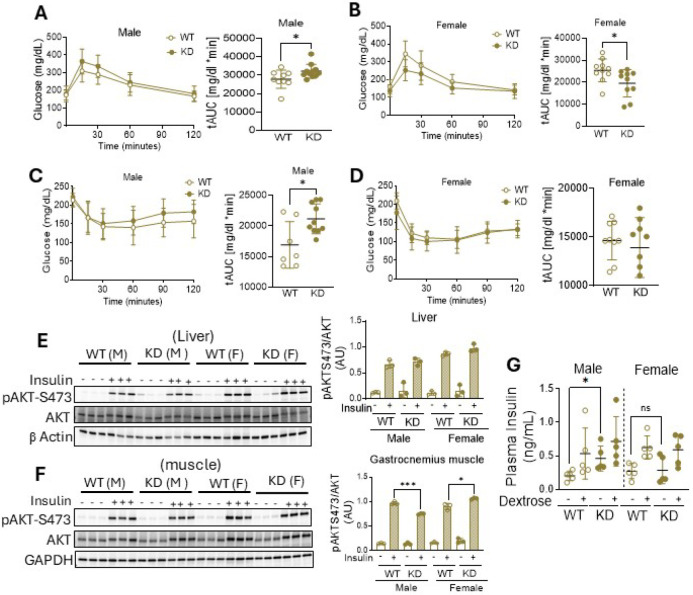
Glycemic control and metabolic parameters of hepatocyte PHB1 deficient mice at 6 months of age. **(A-B)** Blood glucose levels during glucose tolerance test (GTT) of WT and hPHB1-KD male **(A)** and female **(B)** mice with total AUC analysis for each test (n = 6 −12/group). **(C-D)**. Blood glucose levels during insulin tolerance test (ITT) of WT and hPHB1-KD male **(C)**and female **(D)** mice with total AUC analysis for each test (n = 6 −12/group). **(E-F)**. Representative immunoblots of phosphorylated AKT at Serin 473 (S473) in liver **(E)** and gastrocnemius muscle lysates **(F)** from WT and hPHB1-KD male and female mice after fasting for 6 hours followed by i.p. insulin stimulation (10 units/kg) (n = 3/group). **G**. Plasma insulin levels in WT and hPHB1-KD male and female mice after 6 hours fasting followed by i.p. dextrose administration (1g/kg) (n = 5/group). Unpaired t-test was used for comparison between genotypes within the same sex group. All data are presented as mean ± SD. *P < 0.05, ***P<0.001.

Given the established association between hepatic steatosis and loss of glycemic control (24), we next assessed glucose and insulin tolerance in hPHB1-KD mice. Glucose (GTT) and insulin tolerance tests (ITT) revealed that male hPHB1-KD mice exhibited impaired glucose tolerance and reduced insulin sensitivity as indicated by a significant increase in the area under the curve (AUC) for both tests (Fig. 2A, C). Surprisingly, hPHB1-KD females had improved glucose tolerance and no change in insulin responsiveness (Fig. 2B, D). These alterations in glycemic control were not due to changes in gluconeogenesis, as no significant differences were observed with pyruvate tolerance tests in hPHB1-KD mice compared with WT in either sex (Figure S3). To evaluate insulin signaling at the molecular level, a subset of mice was fasted for 6 hours and administered either i.p. insulin (10 U/kg) or saline and euthanized 15 minutes later. Insulin-stimulated Ser473 (S473) AKT phosphorylation was unchanged in livers of hPHB1-KD mice from both sexes compared with WT (Fig. 2E). In gastrocnemius muscle, however, insulin-stimulated phosphorylation was significantly lower in male hPHB1-KD but was unexpectedly enhanced in female hPHB1-KD muscle relative to WT (Fig. 2F). We further assessed glucose-stimulated insulin release by fasting mice for 6 hours followed by i.p. administration of either saline or 50% dextrose (1 g/kg), then euthanized after 30 minutes. Male hPHB1-KD mice displayed elevated basal plasma insulin concentrations, even in the absence of glucose stimulation, while no significant differences in plasma insulin levels were observed among female hPHB1-KD mice (Fig. 2G).

### Transcript-/metabolomic analysis reveals Lpin1 as a novel regulator of lipogenesis and MASLD pathogenesis in male mice with hepatocyte PHB1 deficiency

**Figure 3:**
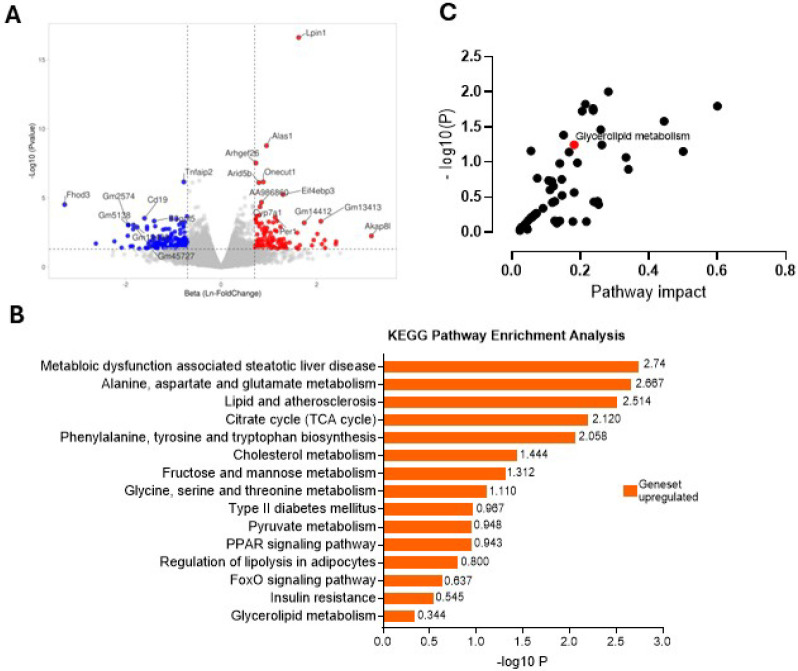
Liver transcript-/metabolomic analysis. **A**. Volcano plot representation of DEG’s in liver of hPHB1-KD males compared with WT. Annotated genes on the plot represent DEG’s that are significantly up- (red) or downregulated (blue) in hPHB1-KD mice. The Y-axis shows statistical significance, expressed as −log10 p-value while the X-axis denotes the beta value (natural log of fold change) between groups. **B**. KEGG pathway enrichment analysis performed using BioJupies software. Bar graph was prepared using GraphPad prism 10 software to show upregulated pathways involved in liver metabolism **C**. Joint pathway analysis by integrating transcriptomic (DEGs with p value <0.05 and beta value > 0.35 (one-fold change)) and metabolomic (metabolites with p-value <0.1) data using KEGG pathway on MetaboAnalyst software version 5. The plot represents the pathway impact with its significance presented as −1og10 p-value.

To gather mechanistic insights on changes at the molecular level in the hPHB1-KD mice, total RNA sequencing and unbiased metabolomic analysis was performed on bulk liver tissue samples which had been immediately clamped in liquid N_2_ – cooled aluminum tongs after rapid dissection to minimize any hypoxia-related changes during tissue harvest (25). Differential gene expression (DEG) analysis identified approximately 20 genes with significantly altered expression between WT and hPHB1-KD male mice, based on adjusted p-value (< 0.05) and fold change > 1 or < −1 (corresponds to beta value (Ln FC) > 0.35 or < −0.35) (Table S1). Intriguingly, of the DEG’s, a ~ 4-fold up-regulation of *Lpin1* was observed in male hPHB1-KD mice compared with WT (adjusted p-value < 0.0005, beta value = 1.623); Fig. 3A; Table S1). *Lpin1* encodes lipin-1, a phosphatidic acid phosphatase that regulates hepatic lipid metabolism at multiple levels and compartmentalized manner in hepatocytes. In the cytoplasm, Lipin1 promotes triglyceride synthesis through its phosphatidic acid phosphatase function, while in the nucleus, it acts as a transcriptional co-regulator to enhance fatty acid oxidation (26). Notably, *Lpin1* up-regulation was not observed in females (Table S2). Subsequent KEGG pathway enrichment analysis, performed using the BioJupies platform, also revealed significant upregulation of the MASLD pathway in male hPHB1-KD mice (Fig. 3B, Table S3), while in female hPHB1-KD mice this pathway was downregulated (Table S4). For deeper insight, we conducted unbiased metabolomic profiling of the liver samples (Table S5), followed by integrative pathway analysis using MetaboAnalyst 5.0 software. DEG’s with p-value < 0.05 in male mice were analyzed in conjunction with metabolites showing a p-value < 0.1 using KEGG pathway. This integrative analysis revealed glycerolipid metabolism as one of the most significantly enriched pathways in hPHB1-KD males compared with WT (Fig. 3C).

### PHB1 knockdown increases cytoplasmic lipin-1 and triglyceride accumulation in hepatocytes

**Figure 4:**
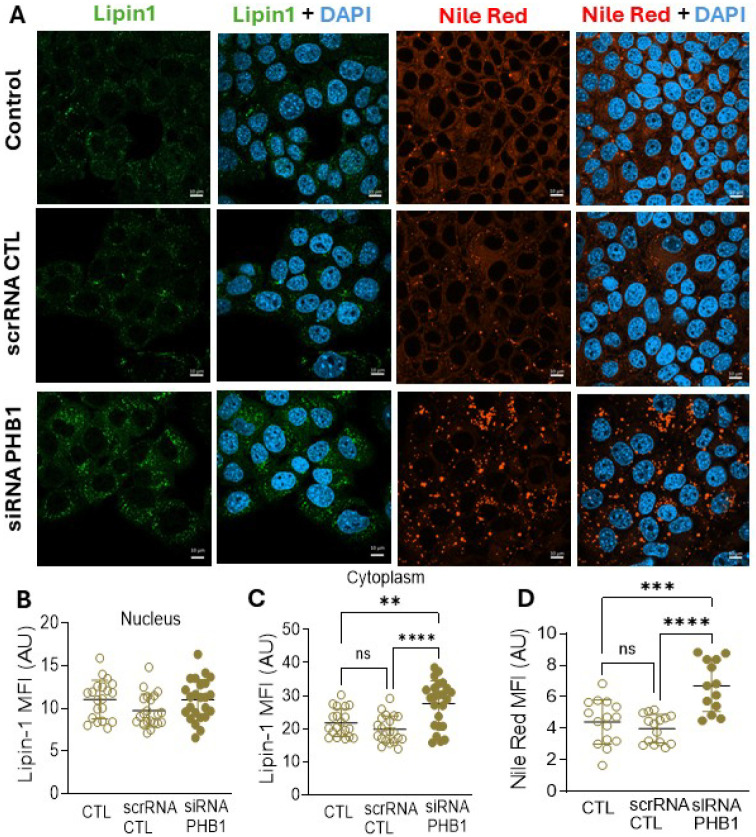
Effect of acute PHB1 knockdown in cultured AML12 hepatocytes. **A**. The left panels depict representative confocal microscopy images of lipin-1 (green) and its overlay with nuclear DAPI stain. The right panels depict representative images for Nile red staining of lipid droplets (red) and the overlay with DAPI. Images were captured at 63X magnification. Scale bar= 10μm. **(B-C)**. Lipin-1 mean fluorescence intensity (MFI) in the nucleus **(B)** and cytoplasm **(C)** expressed in arbitrary units (AU), n=20–24 images/group. **D**. Quantification of mean fluorescence intensity (MFI) of Nile red expressed in arbitrary units (AU), n= 14–16 images/group. Analysis was performed using Zen blue software. Each data point represents one image field captured for that particular group. All data are presented as mean SD. One way ANOVA for three group comparison. **P<0.005, ***P<0.0005, ****P<0.0001.

We next examined lipin-1 content in liver samples from hPHB1-KD and WT mice but found no significant differences in either sex (Figure S4). However, bulk liver tissue is a dense and heterogenous mix of many cell types, so to determine whether PHB1 deficiency specifically affects lipin-1 expression and localization in hepatocytes, we performed targeted silencing of PHB1 in AML12 hepatocytes using siRNA targeting *Phb1* mRNA, or a scrambled non-targeting control siRNA (scrRNA). Immunoblot confirmed *Phb1* siRNA enabled ≥ 50% knockdown of PHB1 compared with scrRNA, consistent with our hPHB1-KD mouse model (Figure S5). Importantly, immunofluorescent analysis revealed that PHB1-deficient AML12 cells had a significant increase in cytoplasmic lipin-1 compared with controls, while nuclear fluorescence levels remained unchanged (Fig. 4A left panels, B-C). Furthermore, lipid droplets stained with Nile Red were substantially increased in the AML12 cells following PHB1 knockdown (Fig. 4A right panels, D).

### Hyper-active mTORC1 signaling underlies pathogenesis of liver steatosis in male mice with hepatocyte PHB1 deficiency

**Figure 5:**
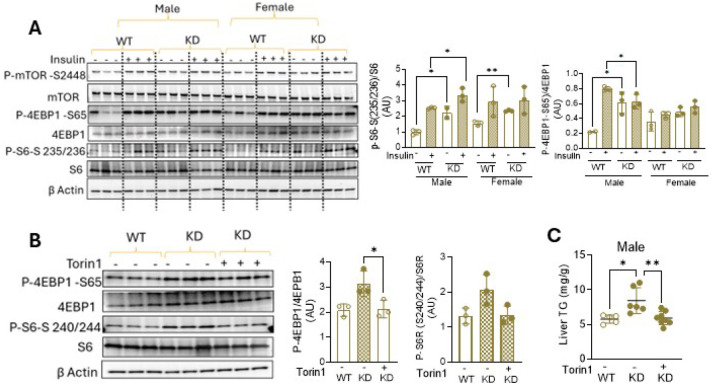
Persistent mTORC1 signaling in hPHB1-KD liver and the effect of Torin1 treatment. **A.** Representative immunoblots for phosphorylated mTORC1 and its downstream targets 4-EBP1 (at Serine 65, S65) and S6 (at Serine 235/236, S235/236) in liver lysates of WT and hPHB1-KD male and female mice (n= 3/group), at baseline and following insulin stimulation (10 units/kg), with corresponding densitometry analysis shown in panels to the right. *p<0.05, **P<0.005. **B**. Representative immunoblots of phospho-4EBP1 and phospho-S6 after 7 days of daily Torin1 (20mg/kg, i.p.) or vehicle treatment in male WT and hPHB1-KD mice (n= 3/group), with corresponding densitometry analysis shown in panels to the right. *p<0.05. C. Quantification of liver triglyceride (TG) content after Torin1 or vehicle treatment in male WT and hPHB1-KD mice. Data are normalized to liver tissue weight (mg/g). N=5–8/group. *P < 0.05, **P<0.01. All data are presented as mean ± SD. Unpaired *t*-test for comparisons.

Prior studies have reported that mTORC1 regulates lipin-1 expression and function in multiple cell types (20, 27, 28), and given the link between PHB’s and mTORC1 implicated in the studies described above, we next tested whether mTORC1 signaling is perturbed in hepatocytes with PHB1 deficiency. Immunoblot analysis of both AML12 (Figure S5) and liver tissues from hPHB1-KD mice clearly show that PHB1 knockdown leads to increased phosphorylation of the well-known mTORC1 targets S6 at Serine 235/236, and 4EBP1 at serine 65 (Fig. 5A). Notably, this activation of mTORC1 signaling in hPHB1-KD liver was not significantly augmented by insulin stimulation in either sex. To determine whether persistent mTORC1 activation plays a mechanistic role in the pathogenesis of hepatic steatosis, a cohort of 22-week-old hPHB1-KD male mice was treated with daily injections of Torin1 (20 mg/kg, i.p.), a potent mTORC1 inhibitor, or vehicle, for seven consecutive days. Torin1 treatment suppressed mTORC1 signaling, corresponding with decreased S6 and 4EBP1 phosphorylation in the liver (Fig. 5B) and effectively reversed hepatic triglyceride accumulation in hPHB1-KD male mice to levels comparable to those of vehicle-treated WT controls (Fig. 5C).

## Discussion

MASLD has become the most prevalent chronic liver disease globally, affecting more than 30% of adolescents and adults (29). It encompasses a pathological continuum ranging from benign hepatic steatosis to progressive stages such as MASH, fibrosis, cirrhosis, and ultimately hepatocellular carcinoma, all of which contribute to considerable clinical and economic burdens (30). Additionally, MASLD, especially MASH with fibrosis, increases the risk of type 2 diabetes, cardiovascular disease, and certain extrahepatic cancers (31, 32). Current therapies for MASLD primarily aim to reduce hepatic lipid burden or enhance their clearance, with several agents showing promise in improving steatosis and inflammation; however, most are limited by side effects and do not address the underlying metabolic dysregulation (33–36). Thus, new targets that drive MASLD pathogenesis are urgently needed for therapeutic investigation.

A comprehensive proteomic study published > 20 years ago reported that PHB1 is downregulated in livers of *ob/ob* mice and obese humans, and in a mouse model of MASH (12). Despite over two decades of work since then, the link between PHB1 downregulation and liver disease pathogenesis has remained largely obscure. In this study, we demonstrated that hepatocyte-specific knockdown (but not complete ablation) of PHB1 leads to hepatic steatosis and impaired glucose tolerance in male mice by 6 months of age, characteristic of MASLD. Mechanistically, hepatocyte PHB1 deficiency induced constitutive mTORC1 activation and upregulated expression and cytoplasmic localization of lipin-1, a key regulator of lipid metabolism in hepatocyte. Importantly, we further demonstrate that the pathological lipid accumulation in hPHB1-KD male mice is, at least in part, mTORC1-dependent, as pharmacological inhibition by Torin1 mitigated hepatic steatosis.

A role for PHB1 in regulating mitochondrial function has been well-characterized (37), and evidence implicating its broader role in metabolic regulation by the liver has emerged in recent years (3). Disruptions in PHB1 expression have been observed during the onset of MASH in a *MAT1A*^−/−^ mouse model as previously described (3, 12), as well as methionine-choline-deficient diet models (10, 38), where steatosis emerged in PHB1 deficient mice as early as 3 days after onset of the diet (10). Our study is the first to report a mechanism by which PHB1 down-regulation in hepatocytes even in absence of injury leads to MASLD development, and document a clear role for aberrant mTORC1 signaling as a major pathogenic driver of the disease. Importantly, hepatocyte PHB1 deficiency causes steatosis and loss of glycemic control in male mice even without a high fat diet or increased caloric intake, underscoring its pivotal role in controlling metabolic homeostasis in liver.

In a previous study, full ablation of PHB1 in hepatocytes caused severe liver injury as early as 3 weeks of age, associated with necrosis, inflammation, and mitochondrial dysfunction, with progression to hepatocellular carcinoma by 8 months. Of note, the mice in that study did not exhibit significant triglyceride accumulation at 14 weeks, despite increased plasma cholesterol (11). Our model of hepatocyte PHB1 haploinsufficiency maintained liver mitochondrial integrity and is consistent with a prior report which demonstrated that partial PHB1 loss in hepatocytes does not disrupt mitochondrial gene expression (39). Furthermore, transcriptomic profiling of both mouse and human liver tissues across various stages of MASLD revealed a weak association between MASLD progression and mitochondrial dysfunction (40). A strength of the partial PHB1 knockdown approach is that it enabled us to investigate the mechanisms by which PHB1 regulates hepatic lipid metabolism independently of liver injury or mitochondrial impairments, both of which are severe confounders.

A key finding of our study is the upregulation of *lpin1* gene in male hPHB1-KD mice. Lipin-1 is a member of the lipin family of proteins, Mg^2+^ -dependent enzymes that function as phosphatidic acid phosphatases in mammalian cells to regulate lipid metabolism via triglyceride synthesis and transcriptional regulation of lipid handling genes. In liver, the function of lipin-1 depends on its cellular localization. In nucleus, it acts as a transcriptional coactivator of PPARα and PGC1α to induce the expression of genes associated with mitochondrial fatty acid oxidation, including suppression of SREBP1 transcriptional activity, thereby decreasing lipogenesis. In cytoplasm, lipin-1 catalyzes the dephosphorylation of phosphatidic acid to produce diacylglycerol, a key step in TG synthesis, and VLDL secretion (28, 41). It was shown that mice with liver *lpin1* ablation had decreased body weight, adiposity, and liver and plasma triglyceride content (41). Our study revealed a sex-specific upregulation of *lpin1* gene expression in livers of hPHB1-KD males, although we did not observe an increase in lipin-1 protein expression in liver lysates from these mice, suggesting that lipin-1 may be regulated at the post-transcriptional level and possibly cell-type specific within the heterogenous tissue of the liver. Since lipin-1 functions as a downstream effector of mTORC1, the increased *Lpin1* gene expression observed in hPHB1-KD males may be mediated through PHB1 effect on mTORC1 signaling specifically in hepatocytes. Prior studies have shown that activated mTORC1 phosphorylates lipin-1 and enhances its cytoplasmic localization (20, 28), an essential control node for sustaining lipin-1 phosphatidic acid phosphatase activity in response to changes in intracellular pH, membrane structure, or fatty acid accumulation. Also, mTORC-1 phosphorylation of lipin1 promotes the nuclear entry of activated SREBP1 and expression of its target lipogenic genes (26). Conversely, treatment with mTORC1 inhibitor, Torin1, promotes the nuclear localization of Lipin-1. This nuclear shift plays a critical role in regulating the SREPB1c transcriptional activity (20, 28, 42). Our transcriptomic analysis showed modest downregulation of Srebpf1 expression (Ln FC= −0.52; ~2-fold change) in the hPHB1-KD male liver, possibly an adaptation to the steatosis.

A noteworthy finding of our study is that the transcriptomic and metabolic alterations seen with mTORC1 activation in the hPHB1-KD male mice were absent in females, indicating a sex-specific effect of hepatic PHB1 deficiency. Previous studies have reported crosstalk between estrogen and insulin signaling in the regulation of *Lpin1* in liver (43). It is therefore plausible that estrogen signaling in females mitigates the upregulation of *Lpin1* associated with PHB1 deficiency, thereby conferring protection against MASLD pathogenesis in female mice.

Our data highlight a male-specific susceptibility to steatosis and systemic insulin resistance associated with PHB1 deficiency, aligning with previous reports that PHB1 interacts with sex hormone signaling pathways (44) and results in sex specific metabolic phenotypes in white adipose tissue and liver (45). In an *in vitro* model, estradiol treatment of 3T3-L1 and C9 cell lines resulted in a dose-dependent increase in both *Phb1* mRNA and protein levels. Furthermore, PHB1 expression was significantly reduced when those cells were co-incubated with the estrogen receptor antagonist fulvestrant, indicating that up-regulation of PHB1 was mediated by estrogen receptor (46). Together with our findings, the evidence points to a potential role for sex steroid signaling in protecting female mice against metabolic dysfunction, warranting further investigation.

mTOR is a serine/threonine kinase that mediates its regulatory functions through two structurally and functionally distinct protein complexes, mTOR complex 1 (mTORC1) and mTOR complex 2 (mTORC2), each characterized by specific components, downstream effectors, and biological functions. mTORC1 drives cellular anabolic processes such as protein and lipid biosynthesis, while mTORC2 is primarily involved in regulating cell survival and cytoskeletal organization (18). Although this study is the first to link PHB1 to mTORC1 signaling in liver, PHB1 has been implicated in regulation of mTORC1 in a few prior studies with disparate cell models, most of which are proliferating cells. In an ovarian cancer cell line, PHB1 was found to inhibit mTORC1 through its interaction with FKBP8 (14). Two other studies have shown that PHB1 deletion leads to persistent upregulation of mTORC1 signaling, which are aligned more closely with our findings (21, 22) which show persistent mTORC1 activation in PHB1-deficient livers even in the absence of insulin stimulation and under fasting conditions. The pathogenic effect of this persistent mTORC1 signaling was revealed by the determination that one week of Torin1 treatment significantly suppressed mTORC1 signaling in liver and attenuated hepatic lipid accumulation in male PHB1 KD mice, suggesting that in normal condition, PHB1 negatively regulates mTORC1 to maintain lipid homeostasis in liver.

Taken together, our findings support a model in which PHB1 regulates hepatic lipid metabolism by modulating mTORC1-dependent control of lipin-1 localization, promoting its nuclear functions and preventing excessive triglyceride synthesis in the cytoplasm.

An important consideration from our model is that PHB1 knockdown caused depletion of PHB2 (Figure S1C), consistent with what is known about the interdependence of these proteins. It is intriguing that hepatocyte-specific PHB2 knockout mice have a number of metabolic features that are directly opposite those of hPHB1-KD mice. Specifically, the PHB2 knockout mice exhibit severe hypoglycemia, impaired insulin signaling, reduced plasma insulin levels, impaired gluconeogenesis, and reduced hepatic glycogen content. Moreover, the hepatocyte-specific PHB2 knockout mice develop hypolipidemia in parallel with liver steatosis, along with a sharp reduction in white adipose tissue mass, indicative of increased lipolysis and fat redistribution to the liver (47). Unfortunately, mTORC1 signaling was not evaluated in the liver from these mice, but it is clear from the divergence in phenotype that although PHB1 and PHB2 are interdependent in their cellular expression, they govern hepatic metabolism through distinct regulatory mechanisms.

To conclude, our study illustrates a clear pathological mechanism linking decreased hepatocyte PHB1 to MASLD via unrestrained and persistent mTORC1 signaling, ultimately leading to increased cytoplasmic lipin-1 expression, steatosis and loss of glycemic control in male mice. Furthermore, the sex-specific nature of this phenotype unmasks PHB’s as potential regulatory nodes of sex signaling, particularly in metabolic disorders and endocrinology. They also reveal PHB’s as provocative therapeutic targets to mitigate MASLD and MASH.

## Materials and Methods

### Animal Model

All animal experiments were approved by the University of Iowa Institutional Animal Care and Use Committee (IACUC). Mice with hepatocyte-specific PHB1-deficienecy were generated by crossing homozygous *Phb1* floxxed (*Phb1*^*fl/fl*^) mice, in which exon 2 of the *Phb* gene is flanked by loxP sites, with Albumin-Cre mice (Alb-Cre). Beginning at 8 weeks of age, male and female WT and hPHB1-KD mice weremaintained on regular chow diet (Research Diets (D20122207), NJ, USA; 12.7% kcal from fat, 72.3% kcal from carbohydrates, and 15% kcal from protein) for a total duration of 16 weeks. Study personnel remained blinded regarding mice genotypes until group assignments were revealed at time of analysis.

### Metabolic testing and phenotyping

Mice body weights were continuously monitored throughout duration of the study. At ~ 5 months of age, mice were subjected to glucose (GTT), pyruvate (PTT), and insulin tolerance (ITT) tests separated by week-long intervals. On the day of each test, mice were singly housed and subjected to a 6-hour food restriction period. For GTT, mice were administered an i.p. injection of dextrose (1 g/kg of a 50% dextrose/saline solution (Nova Tech Inc., NE, USA)). For PTT, mice were administered an i.p. injection of a 1:10 pyruvate: lactate solution (Sigma, WI, USA) equivalent to 2 g/kg pyruvate. For ITT, mice were administered an i.p. injection of 0.75 units/kg of insulin (Eli Lilly, IN, USA). Blood glucose levels were measured from tail vein at 0-, 30-, 60-, and 120-minutes post-injection using a OneTouch Verio Flex glucometer (Lifescan, Switzerland).

Body fat and lean mass were noninvasively measured using NMR within 24 hours prior to euthanasia using the Time Domain NMR Analyzer (minispec LF50, Bruker, MA, USA). Mice were euthanized under isoflurane anesthesia, and tissues were rapidly dissected and freeze clamped using aluminum tongs pre-cooled in liquid nitrogen.

For the Torin1 experiment, male mice at age of 22-weeks received either Torin1 (20 mg/kg) (MedChemExpress, NJ, USA) or vehicle (20% N-methyl-2-pyrrolidone, NMP, 40% PEG400 in water) once daily via i.p. administration for 7 consecutive days. Following the treatment period, mice were euthanized and liver samples were collected for analysis as described above.

### Plasma and liver triglycerides

Plasma and liver triglyceride content were measured using a commercial kit (Cayman Chemical, MI, USA) according to the manufacturer’s protocol.

### Histology

Liver tissues were fixed in 10% formalin, embedded in paraffin for subsequent staining with hematoxylineosin (H&E) and picrosirius red. For lipid droplets staining, formalin fixed liver tissue was embedded in OCT and frozen, sectioned then stained with Oil Red O. Images were captured using Olympus BX63 microscope.

### Plasma insulin

Mice were fasted for 6 hours and euthanized 30 minutes after saline or 50% dextrose injection (1g/kg i.p). Blood was collected in EDTA coated tubes via exsanguination at time of euthanasia and centrifuged at 6000xg for 6 minutes to isolate plasma. Plasma insulin was measured using a commercial insulin rodent chemiluminescence ELISA kit (Alpco, NH, USA).

### Metabolomics

Unbiased metabolomic profiling of bulk liver tissue samples from WT and hPHB1-KD mice was performed using LC-MS, analyzed by MetaboAnalyst 5.0 web-based platform. A more detailed description of the method can be found in the Supplemental Material online.

### RNA Sequencing

RNA was isolated from liver tissue samples (n = 5/group) using the RNeasy Fibrous Tissue Kit (QIAGEN, Maryland, USA). RNA library preparation and sequencing was performed by the genomics division of the University of Iowa Institute of Human Genetics using standardized protocols and Illumina NovaSeq 6000 genome sequencer. Reads were acquired with 200 b.p. paired-end reads with an average depth of ~ 50M reads per sample. Adapter trimmed fastq were aligned using Kallisto (48) to reference transcriptome (“ftp://ftp.ensembl.org/pub/release98/fasta/mus_musculus/cdna/Mus_musculus.GRCm38.cdna.all.fa.gz”). Kallisto index and Kallisto quant were run using default settings, with specific input parameters of 100 bootstraps and 16 threads. Differential expression analysis was performed using Sleuth version 0.30.0 aggregating abundance on Ensemble genes, with a two-step Likelihood Ratio Test and Wald Test (49) Volcano plots were generated using R package VolcanoseR (50). Reads were also aligned with STAR version XX to an index built of “Mus_musculus.GRCm38.98.chr.gtf”. Gene counts were extracted from BAM files using subread version 1.5.2 and FeatureCounts. This count matrix was uploaded to Biojupies (51) for secondary analyses to calculate DEG’s and enrichR pathway analyses.

Raw sequencing reads are deposited into GEO archive ###. Genes were considered differentially expressed between WT and hPHB1-KD mice based on beta value (> 0.35 or < − 0.35) which corresponds to FC > 1 or < −1, and adjusted p value < 0.05.

### Immunoblot analysis

Liver tissue protein extracts were prepared using lysis buffer (50mM HEPES, 150mM NaCl, 1% Triton X-100, 2mM EGTA. 1mM MgCl2, 0.5mM DTT) supplemented with protease and phosphatase inhibitor (Sigma, WI, USA). AML 12 lysates were prepared using commercial RIPA buffer with the same inhibitors. Protein extracts were loaded onto 4–20% polyacrylamide gel (BioRad, CA, USA) for electrophoresis, then transferred to PVDF or nitrocellulose membrane, blocked in 5% bovine serum albumin (BSA) in 1X Tris-based saline with Tween (TBST). The membrane was incubated in a solution of primary antibodies (Table S6) contained in 0.5% BSA in TBST overnight at 4°C. Following wash steps, membranes were incubated in solution of secondary antibodies (HRP conjugated goat anti-rabbit or anti mouse, Jackson Immunoresearch, PA, USA) for 2 hours at room temperature. After another set of wash steps, membranes were incubated in a chemiluminescent substrate (ThermoFisher Scientific Inc, MA, USA), and images were captured using an iBright FL1000 Imaging System (ThermoFisher Scientific Inc., MA, USA). Densitometry analysis was performed using Image J (https://imagej.net/ij/).

### Liver mitochondria isolation and respiratory flux analysis

Liver mitochondria were isolated as previously described (52). Briefly, liver tissue was dissected and rinsed in cold mitochondrial isolation medium (MIM) (300 mM sucrose, 10 mM Na-HEPES, 0.2 mM EDTA, pH 7.2), then thoroughly minced on ice in MIM supplemented with 0.5% BSA and 1mM EGTA. The tissue suspension was homogenized using a Dounce apparatus, then centrifuged at 600*g* for 10 minutes at 4°C. The supernatant was collected and centrifuged at 8000*g* for 15 minutes at 4°C. The pellet was resuspended in the same buffer and centrifuged again at 8000*g* for 15 minutes at 4°C. The final pellet was resuspended in MIM buffer supplemented with 0.5% BSA and 1mM EGTA. Protein concentration was measured using Pierce BCA protein assay (ThermoFisher Scientific Inc, MA, USA). Mitochondrial respiration was assessed using the Oroboros O_2_K as follows: Briefly, 250 μg of isolated mitochondria were added to Buffer Z-lite (105 mM K-MES, 30 mM KCl, 10 mM KH_2_PO_4_, 5 mM MgCl_2_-6H_2_O, 0.05% BSA, pH 7.4), supplemented with 1 mM EGTA, 4 mM D-glucose, 1.7 U/mL glucose-6-phosphate dehydrogenase and 3.4 U/mL hexokinase (Roche, Sigma, WI, USA). After stabilization of oxygen concentration, substrates were added to measure oxygen consumption in the following order and concentrations, 25 μM palmitoyl carnitine with 2 mM malate, 650 μM ADP, 5 mM glutamate, 5 mM succinate. Data was analyzed using Oroboros DatLab software.

### AML12 culture and Phb1 silencing

AML12 cells were purchased from ATCC (CRL-2254, VA, USA) and cultured in DMEM-F12 supplemented with 10% FBS, 1X insulin transferrin selenium (ITS) and 40 ng/ml dexamethasone and incubated at 37°C with humidified air containing 5% CO2. The siRNA targeting mouse *Phb1* (ON-TARGETplus mouse *Phb* smart pool, L-060391-01-0005) and the nontargeting siRNA (ON-TARGETplus non-targeting pool, D-001810–10-05) were purchased from Horizon Discovery (Horizon Discovery, CO, USA). The non-targeting scrambled siRNA was used as a negative control. AML12 cells were grown in a 24 well plate and reverse transfected with 10nM siRNA using Lipofectamine RNAimax and opti-mem in the complete cell growth medium for 72 hours prior to biochemical analysis and/or imaging.

### Nile red staining of AML12 cells

AML12 cells were washed with PBS twice, fixed in 4% PFA at room temperature for 30 minutes, permeabilized with 0.1% Triton-x100 for 5 minutes, then stained with Nile Red (1 μg/ml; Thermo-Fisher Scientific Inc, MA, USA) for 20 minutes at 37°C. Nuclei were counterstained using VECTASHIELD^®^ anti-fade mounting medium plus DAPI. Images were captured using a Zeiss LSM 980 confocal microscope. Mean fluorescence intensity was analyzed using Zen Blue 3.3 software.

### Immunofluorescence staining of AML12 cells

AML12 cells were washed with PBS, fixed with 4% PFA for 30 minutes at room temperature, permeabilized with 0.1% Triton X-100, and blocked with 5% BSA, and 5% normal goat serum in PBS for 1 hour at room temperature. Subsequently, cells were incubated overnight at 4°C with an anti-lipin1 antibody (Abcam, MA, USA; dilution 1:200). After thorough washing with 0.04% TBST, cells were incubated with Alexa Fluor 488 conjugated secondary antibody (1:200 dilution) for 2 hours at room temperature in the dark. Finally, the cells were mounted using Vectashield antifade mounting medium containing DAPI. Images were captured using a Zeiss LSM 980 confocal microscope. Mean fluorescence intensity was analyzed using Zen blue 3.3 software.

### Statistical analysis

Statistical analysis was performed in GraphPad Prism version 10. Data are presented as mean ± S.D. Unpaired t-test was used for two group comparisons, and one-way ANOVA was used for multiple group comparisons. Alpha-level of significance for main effects between groups was set at P < 0.05.

## Supplementary Material

Additional supplementary information for this article is available online at

Supplementary Files

This is a list of supplementary files associated with this preprint. Click to download.

- SupplementalmethodsDetailedmetabolomics.docx
- OriginalFullLengthWesternBlots.pptx
- SupplementaryFiguresFINAL.pptx
